# Using Osmotic Pumps to Induce the Production of Gametes in Male and Female European Eels

**DOI:** 10.3390/ani12030387

**Published:** 2022-02-05

**Authors:** Marta Blanes-García, Pablo García-Salinas, Marina Morini, Luz Pérez, Juan F. Asturiano, Victor Gallego

**Affiliations:** Grupo de Acuicultura y Biodiversidad, Instituto de Ciencia y Tecnología Animal, Universitat Politècnica de València, Camino de Vera s/n, Edificio 7G, 46022 Valencia, Spain; marblaga@posgrado.upv.es (M.B.-G.); pau.salinas82@gmail.com (P.G.-S.); marina.morini@gmail.com (M.M.); mlpereig@dca.upv.es (L.P.); jfastu@dca.upv.es (J.F.A.)

**Keywords:** European eel, spermiation, ovulation, human chorionic gonadotropin hormone, carp pituitary extract, hormone release system

## Abstract

**Simple Summary:**

The European eel is a species with high commercial value for aquaculture, and it has suffered a drastic reduction of its natural stocks during the last decades; thus, breeding in captivity is nowadays considered essential to avoid the extinction of the species. In this sense, a new method to maturate the European eel (males and females) for eel aquaculture has been studied. For the first time in the European eel, a controlled release hormone system (osmotic pumps) has been tested, which allowed to induce the testis maturation and sperm production in part of the males as well as a very early maturation and ovulation in the females.

**Abstract:**

The European eel (*Anguilla anguilla*) is a commercially valued species for aquaculture. Over the past decades, it has experienced a drastic reduction in its natural stocks. Thus, breeding in captivity is considered essential, nowadays, to guarantee the eel aquaculture and to reduce pressure on natural populations. Traditionally, the European eel has been sexually matured by means of weekly hormonal injections, which cause stress to the fish. The purpose of this research study was to assess the use of osmotic pumps as a new method to induce sexual maturation in male and female European eels, without the weekly injection. The control groups were treated with weekly hormone injections (recombinant human chorionic gonadotropin for males and carp pituitary extract for females), and the implanted groups were treated with osmotic pumps (ALZET^®^ osmotic pumps) loaded with the respective hormones. Regarding male European eels, this study shows that the use of controlled release systems was able to induce the maturation and spermiation, but without the necessary capacity to produce enough gametes with acceptable quality parameters that could meet the needs of a commercial eel hatchery. Concerning female European eels, the study demonstrates that the use of osmotic pumps loaded with CPE became an effective method, generating early maturations (4 to 10 weeks) in 50% of the females, so this method could become a viable alternative for eel hatchery procedures.

## 1. Introduction

The European eel (*Anguilla anguilla*) is a commercially valued species, especially for the Japanese and European markets. Nevertheless, it is not yet possible to breed European eels in captivity, and current aquaculture production consists of fattening wild-caught glass eels. Over the past 50 years, populations of the European eel have been declining, and the recruitment of glass eels has collapsed since the early 1960s [1]. Overfishing, habitat reduction, pollution, and the swimbladder parasite (*Anguillicola crassus*) are some of the causes that have led to the decline of the European eel [2]. As a result, it was included in the Red List of the International Union for Conservation of Nature (IUCN) as a “Critically Endangered” species [3], and measures for the recovery of the stock have been established by the European Union (Regulation 1100/2007, 18 September 2007) [4].

Nowadays, controlling the reproduction and ability to produce glass eels seems to be the only sustainable solution to reduce the pressure on natural populations. However, the complexity of its reproductive physiology makes it difficult to increase the supply of glass eels for aquaculture. The European eel is a catadromous fish with a very complex life cycle, which includes two transoceanic migrations. In order to overcome the lack of natural spawning stimuli in captivity, the sexual maturation of both males and females must be induced with long-term hormonal treatments [4,5,6].

Regarding male European eels, human chorionic gonadotropin (hCG) has been the most widely used hormone for reaching spermiation, but it has been administered to the animals in different formats [7,8]. Currently, the use of weekly injections of recombinant human chorionic gonadotropin (hCG_rec_) is considered an effective alternative and showed better results than hCG in terms of sperm quantity and quality [9,10].

With regard to female European eels, the traditional method consists of weekly injections with carp (CPE) or salmon (SPE) pituitary extracts at around 15 to 20 weeks [5,7]. When the oocytes reach the nuclear migration stage, a final treatment is administered using a dose of the maturation-inducing steroid (DHP) [11,12]. There are several studies on fertilization and hatching trials in the European eel, but the fact is that the individual response of females to treatments is highly variable and spawning percentage, egg quality, or larval hatching is affected [13,14].

Despite the positive results obtained with the traditional hormonal treatments to induce the sexual maturation of the eels in both sexes, these are time-consuming processes, and the weekly injections of hormones require repetitive handling of the broodstock, causing stress, and susceptibility to the diseases [15,16]. In this sense, the study of hormonal pretreatments [17,18], new hormonal treatments, and hormone delivery systems that reduce the level of stress in fish during the induction of the sexual maturation, have been carried out to optimise the protocols for this species. Therefore, over the past years, several gonadotropin-releasing hormone delivery systems have been assessed, including osmotic pumps [19,20]. The mechanism of operation of the osmotic pumps is based on the osmotic difference between the inside of the pump (osmotic layer) and its environment (animal tissue). These delivery devices cause the sexual maturation of the fish by steadily releasing hormones, reducing stress in the fish by avoiding the weekly injections. In this way, the osmotic pumps have been used in several species of teleosts, but the effectiveness was quite variable (*Chanos chanos*: [21]; *Lates calcarifer:* [22]; *Clarias batrachus* and *C. gariepinus:* [23]). The use of osmotic pumps to induce sexual maturation in eels was studied for the first time by Kagawa et al. [24]. It was shown that the implantation of an osmotic pump loaded with hCG stimulates spermatogenesis and spermiation in male Japanese eels (*Anguilla japonica*). Subsequently, the osmotic pumps were used to induce the maturation of captive female Japanese eels. Pumps were loaded with SPE, hCG, and GnRHa, and SPE had better results compared with the other hormones [25].

Therefore, the aim of the present study was to evaluate these hormone delivery systems as an alternative method to induce the sexual maturation of male and female European eels. The production and quality of gametes from both males and females of the different maturation methods were recorded.

## 2. Materials and Methods

### 2.1. Fish Handling

Thirty male eels (mean body weight = 126.7 ± 17.9 g) from the fish farm Valenciana de Acuicultura, S.A. (Puzol, València; Spain) and twenty-one female eels (mean body weight = 771 ± 123.8 g) caught by local fishermen in the Albufera Lagoon (Valencia) during their migration to sea, were moved to our facilities in the Aquaculture Laboratory of the Universitat Politècnica de València, Spain. Each individual eel was tagged with passive integrated transponders (PIT tags) in order to identify the eels. Male eels were distributed into three 150-L aquaria equipped with separate recirculation systems and thermostats/coolers. Female eels were distributed into four 500-L aquaria equipped with IRTAmar^®^ technology [26]. The fish were gradually acclimatised for 1 week from freshwater to sea water (salinity 37 ± 3 g/L). The fish were fasted during the experiment and the aquaria were covered to maintain constant shade (photoperiod: 0L:24D).

This study was carried out in strict accordance with the recommendations given in the Guide for the Care and Use of Laboratory Animals of the Spanish Royal Decree 53/2013 regarding the protection of animals used for scientific purposes (BOE 2013). The protocol was approved by the Experimental Animal Ethics Committee from the Universitat Politècnica de València (UPV) and final permission (2019/VSC/PEA/0034) was given by the local government for managing endangered fish species (Generalitat Valenciana).

### 2.2. Males

#### 2.2.1. Induction of Sexual Maturation: Experimental Design

**Figure 1:**
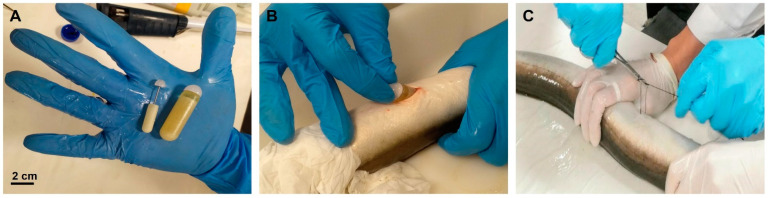
(**A**) Size of the osmotic pump. Scale bar: 2 cm. (**B**) Implantation process of the osmotic pump into the peritoneal cavity. (**C**) Suture of the wound.

The male eels were divided into three experimental groups: control, OP-100, and OP-200 (*n* = 10 per group). Over 15 weeks, the control group was weekly anesthetised with benzocaine (60 ppm) and received an intraperitoneal injection of hCG_rec_ (Ovitrelle^®^, 1.5 IU/g fish). In parallel, the eels from OP-100 and OP-200 groups were anesthetised with benzocaine (60 ppm) and an osmotic pump (ALZET^®^ OsmoticPumps [27]; Figure 1A) was implanted into the peritoneal cavity of each eel after cutting the abdomen wall with a scalpel approximately 0.5 and 2 cm, respectively (Figure 1B). Finally, the wound was sutured (Figure 1C). The control group fish underwent the same surgery and a sterile object of similar size to the osmotic pump was introduced into their intraperitoneal cavities.

The OP-100 group of males received the osmotic pump ALZET^®^-1004 (diameter = 6 mm, length = 15 mm, reservoir volume = 100 μL), while the OP-200 group of fish received the osmotic pump ALZET^®^-2006 (diameter = 7 mm, length = 30 mm, reservoir volume = 200 μL). To load the osmotic pumps, hCG_rec_ was diluted in a saline solution (NaCl 0.9%) at a concentration of 13 IU/μL. In accordance with the instructions of the manufacturer, the ALZET^®^-1004 and ALZET^®^-2006 osmotic pumps can, respectively, release 0.05 and 0.07 μL of the solution per hour for approximately 10 weeks when the fish are maintained at a water temperature of 21 °C. These data were considered to calculate the volume of diluted hormone contained in each type of pump to guarantee that the three groups received a similar amount. After 10 weeks, the osmotic pumps stopped releasing hormone and the eels from the OP-100 and OP-200 groups received weekly intraperitoneal injections of hCG_rec_ (1.5 IU/g fish) for 5 weeks to complete the treatment.

#### 2.2.2. Sperm Collection and Sampling

Before sperm collection, the eels were anesthetised with benzocaine (60 ppm). Sperm samples were collected weekly through the application of abdominal pressure 24 h after the administration of the hormone in the control group (following the protocol described by Pérez et al. [28]), taking special care to avoid contamination with faeces, urine, and sea water. Sperm volume was measured using graduated tubes and sperm density was determined by computer-assisted sperm analysis (CASA) systems (see the next section). After that, samples were diluted 1:24 (sperm:extender) in P1-medium [29] and kept in plastic tubes at 4 °C until the sperm kinetic analysis, which was carried out in the 2 h following sperm collection.

#### 2.2.3. Sperm Motility Assessment

Samples were activated by mixing 0.5 μL of P1-diluted sperm with 4.5 μL of artificial sea water (Aqua Medic, Meersalz, 37 g/L, with 2% BSA (*w*/*v*), pH adjusted to 8.2). All the motility analyses were performed in triplicate using the module of ISAS ^®^ v1 (Proiser R+D, S.L.; Paterna, València, Spain), which uses CASA technology to evaluate the sperm. Video sequences of 0.5 s were recorded (at 60 fps) using a video camera (Nikon Digital Sight DS-5M) mounted on a phase-contrast microscope (Nikon Eclipse 80i) with a 10× objective lens. The chamber used throughout all the analysis was a SpermTrack-10^®^ (Proiser, Paterna, València, Spain) with a 10× negative contrast phase lens on a Nikon Eclipse (E-400) microscope.

The parameters considered in this study were density, defined as the number of spermatozoa/mL; total motility (MOT, %), defined as the percentage of motile cells after sea water activation; and progressive motility (pMOT, %), defined as the percentage of spermatozoa which swim forwards in 80% of a straight line. Spermatozoa were considered motile if their straight-line velocity (VSL) was >10 μm/s.

#### 2.2.4. Biometric Parameters

At the end of the 15-week experimental period, all males were euthanised and weighed. In order to evaluate the progression of maturation, some biometric parameters such as gonadosomatic index [GSI = (gonad weight/total body weight) ∗ 100] and pectoral fin colour (Black, Dark grey, Light grey, Transparent; [30]) were evaluated.

### 2.3. Females

#### 2.3.1. Induction of Sexual Maturation: Experimental Design

The female eels were divided into two experimental groups: the control group (*n* = 11) and the OP-2ML4 group (*n* = 10). The control group was treated with weekly intraperitoneal injections with carp pituitary extract (20 mg/kg; CPE, Catvis, Ltd., The Netherlands) for 15 to 25 weeks (or until ovulation happened). The CPE was diluted in a saline solution (NaCl 0.9%) to obtain a concentration of 0.1 g CPE/mL. The mix was centrifuged (3000 rpm, 10 min.) and the supernatant was stored at −20 °C until use. Once a week, the eels were anesthetised with benzocaine (60 ppm) and weighed before the injections to calculate the individual CPE dose [13]. 

In the OP-2ML4 group, osmotic pumps Alzet-OP-2ML4 (diameter = 14 mm, length = 51 mm, reservoir volume = 2000 μL) were used as the hormone delivery system. The osmotic pumps were implanted in a similar way to that previously described for the males (Section 2.2.1.), by cutting the abdomen wall with a scalpel approximately 0.5 cm. The control group fish underwent the same surgery and a sterile object of similar size to the osmotic pump was introduced into their intraperitoneal cavities.

The osmotic pumps were loaded with the supernatant of CPE. In accordance with the instructions of the manufacturer, the used osmotic pumps can release 1.05 μL of the solution per hour for approximately 10 weeks when the fish are maintained at a water temperature of 20 °C. The volume of diluted CPE contained by the pumps was calculated to contain a similar amount of hormone than the received by the control group. After 10 weeks, the osmotic pumps stopped releasing hormone and the eels from the OP-2ML4 group received weekly intraperitoneal injections of CPE (20 mg/kg fish) for 15 weeks to complete the treatment.

#### 2.3.2. Sexual Maturation Assessment and Spawning Induction

The females were monitored on a weekly basis and the eels that seemed to be sexually mature (based on body weight increase and enlarged abdomen [11]) were moved to a separated tank to follow the maturation evolution over the following days. If the abdomen was quite large, a sample of oocytes was collected by intraovarian cannulation (cannula diameter = 1.6 mm). The oocytes were diluted in saline solution (NaCl 0.9%) and observed using the camera (Nikon Digital Sight DS-5M) mounted on the binocular loupe (Leica MZ16F). If the oocytes were in the 3rd or 4th phase of nucleus migration [11], the female was led to the final maturation stage, following the protocol described by Butts et al. [12]. Consequently, females received a final CPE injection (prime dose 20 mg/kg fish) and 24 h later, if the oocytes were in the 5th phase of development, the females were treated with 17α,20β-dihydroxy-4-pregnen-3-one (DHP) at 2 mg/kg fish. Over the following 12 h, the females were monitored until the spawning occurred. The ripe females were isolated in a separated tank and anaesthetised with benzocaine (60 ppm) and the genital area was cleaned with freshwater before abdominal stripping. The eggs were collected into laboratory trays and weighed to calculate the amount of sperm to be added for in vitro fertilization, using a sperm to egg ratio of a minimum of 1:25,000, according to Butts et al. [12].

#### 2.3.3. Biometric Parameters: Gonadosomatic Index (GSI)

At the end of the 25-week experimental period, the females that did not ovulate during the experiment were euthanised and weighed. To evaluate the progression of maturation, the gonadosomatic index was calculated for females that did not ovulate.

#### 2.3.4. Fertilization, Hatching Rate, and Embryonic Development

Two hours before spawning, sperm from three or four males was collected (total motility >70%) and its quality was determined using the protocol and the CASA system previously mentioned. Sperm (diluted 1:99 in P1-medium) was first mixed with the eggs and then immediately activated with artificial seawater (37 g/L). After 5 min of gamete contact time, eggs were moved for incubation with sterile sea water (19 to 21 °C). During the egg incubation, the dead eggs were removed, and sea water renewals were carried out. Fertilization rate was determined between 4 to 6 h post-fertilization (hpf), by examining a sample of 80 to 100 eggs of each spawn with a FullHD camera (Moticam 1080) mounted on a binocular loupe (Leica MZ16F). Embryonic development was evaluated over the following 48 h.

### 2.4. Statistical Analysis

The mean ± standard error was calculated for all the parameters. Shapiro–Wilk and Levene tests were used to check the normality of data distribution and variance homogeneity, respectively. One-way ANOVA and Kruskal–Wallis tests were used to analyze the sperm quality parameters between groups at the same week. Significant differences between treatments were detected using the Student–Newman–Keuls (SNK) test. Differences between the gonadosomatic indexes in control group and OP groups for both males and females were analyzed using Student’s t-tests (for parametric data) and Mann–Whitney U tests (for non-parametric data). Significant differences were detected when *p*-value < 0.05.

All statistical analyses were performed using the statistical package SPSS version 24.0 for Windows software (SPSS Inc., Chicago, IL, USA).

## 3. Results

### 3.1. Males

#### 3.1.1. Sperm Production: Spermiating Males, Sperm Volume, and Sperm Density

**Figure 2:**
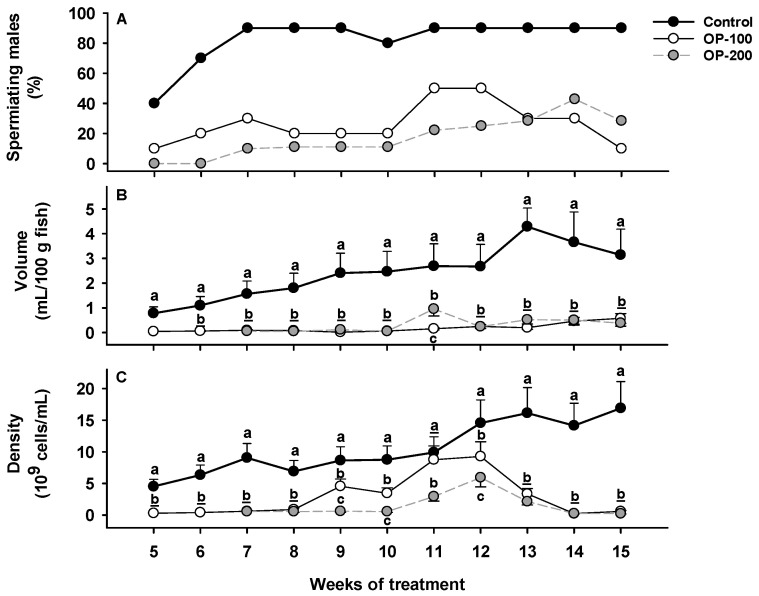
Evolution of sperm production parameters throughout the hormonal treatments (control, OP-100, OP-200). (**A**) Percentage of spermiating males. (**B**) Sperm volume. (**C**) Sperm density. Data are expressed as mean ± SEM. Different letters mean significant differences between treatments at each week of treatment.

Regarding the sperm production, the control group showed the highest percentage of spermiating males (with motile cells) (Figure 2A), achieving values >80% from the 7th week of treatment. The group OP-100 showed more spermiating males than the group OP-200, reaching a value of 50% between the 11th and 12th weeks. In addition, males from the OP-200 group did not produce sperm until the 7th week of the treatment, but males from the control and OP-100 groups produced sperm from the 5th week. 

Regarding sperm volume, the control group showed significantly higher values during the whole spermiation period (Figure 2B), reaching a maximum value of 4 mL/100 g fish at the 13th week of treatment. The OP-100 and OP-200 fish showed similar results with each other during the full period, and significant differences between both groups were found only at the 11th week because the OP-200 group achieved a maximum value of 1 mL/100 g fish.

Concerning the sperm density (Figure 2C), the control group showed higher values than OP groups. The OP-100 and OP-200 groups showed an increase that started earlier (9th week) and reached higher values in the OP-100 group, reaching the maximum value in the 12th week, while the control group did it in the 15th week (>15 × 10^9^ spermatozoa/mL).

#### 3.1.2. Sperm Quality Assessment: Total Motility and Progressive Motility

**Figure 3:**
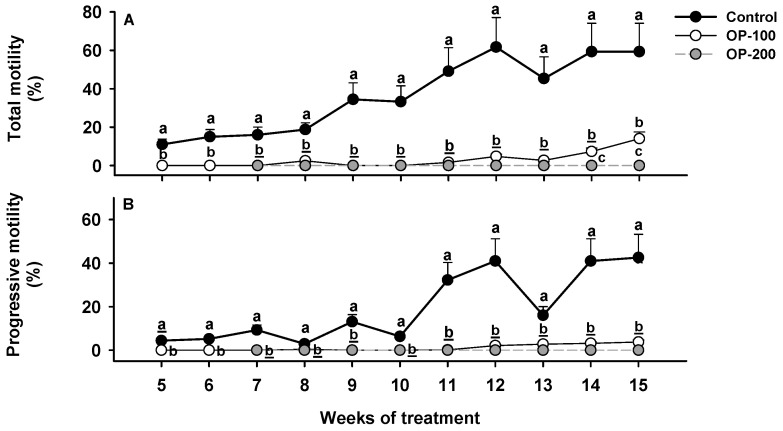
Evolution of sperm motility parameters throughout the hormonal treatments (control, OP-100 and OP-200). (**A**) Percentage of motile cells. (**B**) Percentage of progressive motile cells. Data are expressed as mean ± SEM. Different letters mean significant differences between treatments at each week of treatment.

Regarding sperm motility patterns (Figure 3A,B), the control group showed higher values than experimental groups, reaching maximum MOT values of 61.7% and maximum pMOT values of 40.9% in the 12th week. The OP-100 and OP-200 groups maintained lower MOT and pMOT values than 15 and 5%, respectively, throughout the treatment.

#### 3.1.3. Biometric Parameters

**Figure 4:**
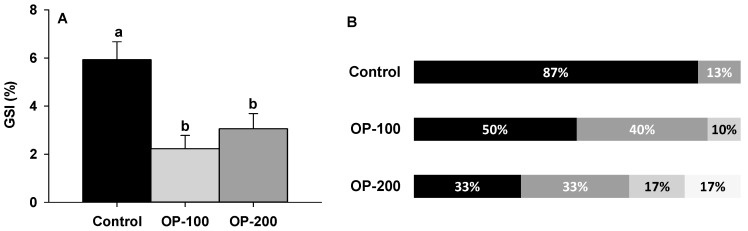
(**A**) Gonadosomatic index at the end of the experiment (week 15) on the control, OP-100, and OP-200 groups. (**B**) Percentage of fish showing pectoral fin colour (black, dark grey, light grey, transparent) at the end of the experiment (week 15) on the control, OP-100, and OP-200 groups. Data are expressed as mean ± SEM. Different letters mean significant differences between treatments.

Regarding the mean GSI values (Figure 4A), a significant difference was found between groups, where control (5.92 ± 1.02) showed higher values than experimental groups OP-100 (2.23 ± 0.81) and OP-200 (3.06 ± 1.43). 

Concerning the pectoral fin colour (Figure 4B), it was found to be different in shading, which is considered a maturation biomarker [31]. In sexually immature eels, the pectoral fin is transparent, and it becomes darker during the sexual maturation. Prior to the experiment, the eel fins showed a transparent colour. At the end of the experiment, 87% of the control group males showed a black fin that was present in only 50% and 33% of the males in the OP-100 and OP-200 groups, respectively. A dark grey fin was found only in 13% of the control group, but in 40% and 33% of OP-100 and OP-200 groups, respectively.

**Figure 5:**
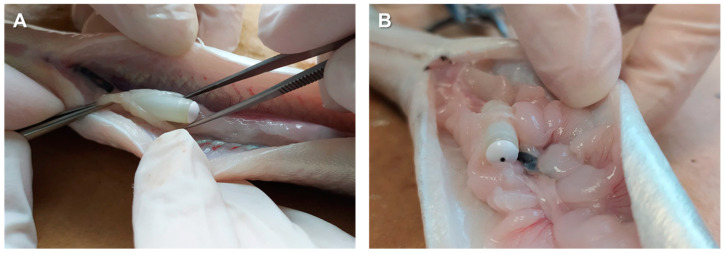
(**A**) Osmotic pump encapsulated in the conjunctive tissue of a male European eel. (**B**) Osmotic pump implanted in the peritoneal cavity of a male European eel.

In the osmotic pump groups, there were animals that did not show any signs of maturation. In this sense, some of these males had the osmotic pump strongly encapsulated inside the conjunctive tissue (Figure 5A). However, males that showed gonadal maturation in the osmotic pump groups presented the osmotic pump free along the peritoneal cavity (Figure 5B).

### 3.2. Females

#### 3.2.1. Sexual Maturation Evaluation

Regarding the percentage of females that responded to the hormonal treatments, 10 out of 11 females from the control group and 8 out of 10 females from the OP-2ML4 reached the ovulation stage. Regarding the spawning induction, six females were induced to a spawning event and subject to abdominal stripping.

#### 3.2.2. Weight Evolution during Sexual Maturation

**Figure 6:**
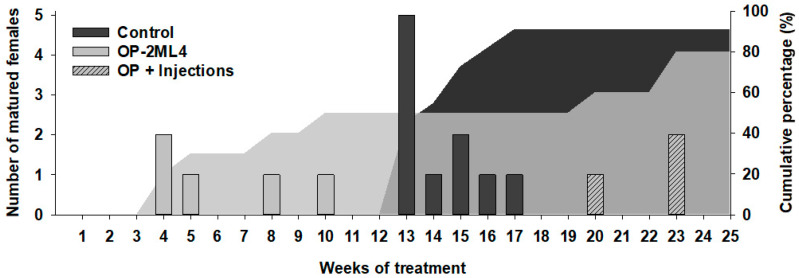
Bars show the number of mature females in each week of treatment (control and OP-2ML4), and shaded area shows the cumulative percentage of mature females. Bars in the OP+injections group correspond to the eels in the OP-2ML4 group that continued receiving injections until week 25 of the experiment.

**Figure A1:**
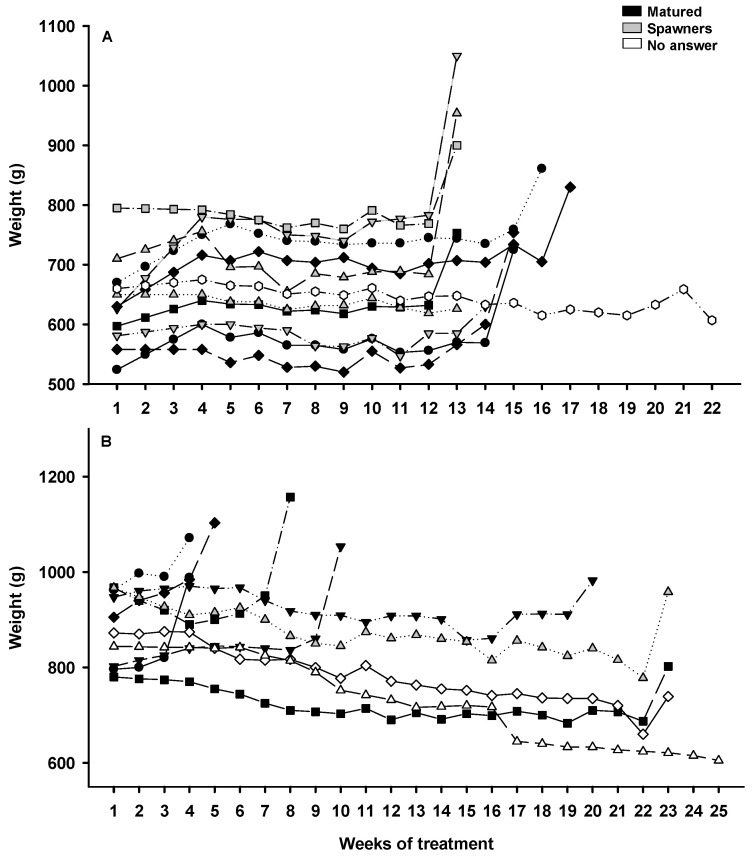
Weight evolution (%) regarding the initial weight of each female from (**A**) control group and (**B**) OP-2ML4 group over the weeks of treatment.

Altogether, all the females from the control and the OP-2ML4 groups that responded to the hormonal treatment showed a weight increment in the weeks prior to sexual maturation (reaching means of 20 ± 14 and 16 ± 6%, respectively; see Appendix A, Figure A1; females that did not respond to the treatment exhibited a weight decrease). Regarding the time of maturation, the females from control group matured after 13 to 17 weeks of treatment, while females in the OP-2ML4 group showed two maturation phases: an early phase (females matured after the 4th to 10th week) and a late phase (20th to 23rd week) (Figure 6). A female in the OP-2ML4 group was able to show two ovulations: the first occurred in the 4th week and the second in the 20th week.

**Figure 7:**
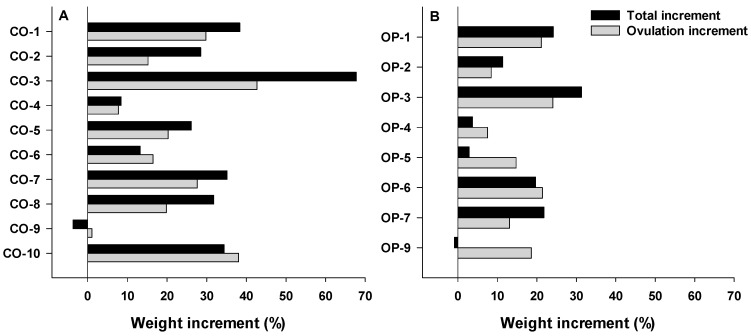
Total weight increase (%, black) comparing the body weight at the maturation week and the body weight at the beginning of the experiment; and Ovulation weight increase (%, grey) comparing the body weight at the maturation week and the body weight from the previous week, of each female from (**A**) control group and (**B**) OP-2ML4 group. Females that did not respond to the treatments are not shown.

There were differences in the weight increase in both groups, with respect to their initial weights. The control group showed a mean increase of 28 ± 20%, with a range between 8% and 68% (Figure 7A). The OP-2LM4 group showed a mean increase of 16 ± 14%, reaching a maximum value of 36% and a minimum value of 3% (Figure 7B).

#### 3.2.3. Biometric Parameter: Gonadosomatic Index (GSI)

**Figure 8:**
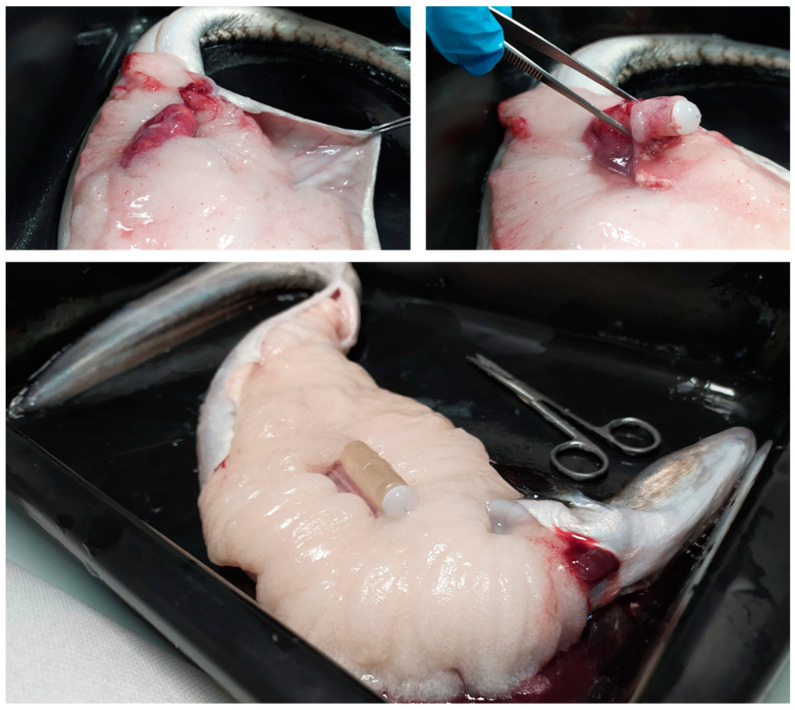
Process of extraction of an osmotic pump from the peritoneal cavity of a matured female European eel.

Both control and OP-2ML4 groups showed similar results, showing ovulation and gonad development (Figure 8). The control group reached a GSI of 51.2 ± 9.8%, while the OP-2ML4 group had a mean value of 49.5 ± 2.8%.

#### 3.2.4. Fertilization, Hatching Rate, and Embryonic Development

**Figure A2:**
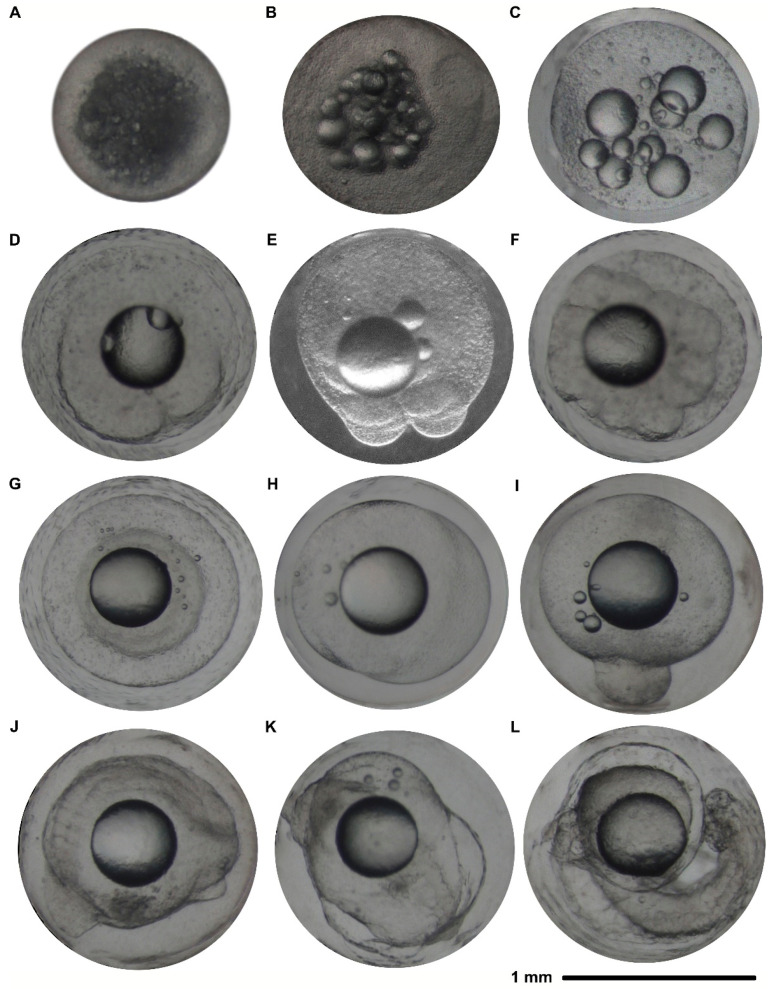
Oocyte developmental stages of European eel. (**A**) Immature oocyte after extrusion by stripping. (**B**) Oocyte in state V (when the DHP injection is given to induce ovulation). (**C**) Perivitelline space formed (0 h 25 min post-fertilization). (**D**) Two-cell stage (5 h 30 min p.f.). (**E**) Four-cell stage (6 h 20 min p.f.). (**F**) Morula stage (6 h p.f.). (**G**) Gastrula stage, formation of germ ring (6 h 50 min p.f.). (**H**) Epiboly stage, 50% (8 h p.f.). (**I**) Blastopore closure (15 h 30 min p.f.). (**J**) Segmentation state (30 h 15 min p.f.). (**K**) Embryo formed, eye vesicle and somites along most of the embryo (32 h p.f.). (**L**) Embryo has clearly distinguishable head, eyes, and somites (33 h p.f.). Scale bar: 1 mm.

At the end of the experiment, the eggs collected from the six spawning females (five from control group and one from the OP-2ML4 group) were incubated to evaluate their embryonic development (see Appendix A, Figure A2). Embryos survived up to 33 h post-fertilization, and no hatching was observed.

## 4. Discussion

The traditional hormonal treatments to induce sexual maturation in fish entails a continuous handling of the animals, which has a negative impact on the maturation process due to the stress suffered by the fish. As a result, in over the past decades sustained-release delivery systems have been used, principally to control oocyte maturation in females, but also to improve the spermiation in several species. There are different sustained-release delivery systems available, such as cholesterol pellets, ethylene-vinyl acetate implants, or biodegradable microspheres, which have been widely used to control the reproduction in fishes [19]. The osmotic pumps have shown positive results in several fish species (*Gadus morhua,* [32]; *Clarias gariepinus* [23,33]), including the Japanese eel [24,25]. To date, the efficacy of osmotic pumps to induce the sexual maturation has not been proven in the European eel.

### 4.1. Males

This study shows that the osmotic pumps (ALZET; OP-100 and OP-200) can induce the testis maturation and sperm the production in part of the males.

However, the sperm volumes produced by these males were significantly lower compared to the control group. The results of control group were similar to those obtained by Herranz-Jusdado et al. [10], since, in both studies the males started to produce sperm in the 6th week of the treatment and with similar volumes and densities throughout the weeks of experiment. Additionally, the low-density values of the OP-100 and OP-200 groups were not enough to make up for the low volume values. 

In addition to density, sperm quality is crucial to fertilization trials. In several fish species, the fertilization and hatching rates are used to evaluate the sperm quality, but due to the reproduction limitations of the European eel, quality is assessed by kinetic parameters (motility and velocity) of the spermatozoa [34]. In the present study, the spermatozoa from the OP-200 samples never showed motility during the experiment. However, the samples from the OP-100 group showed motility from the 10th week and reached a maximum (around 20% of motile cells) in the 15th week. In previous studies, Kagawa et al. [24] tried several doses of hormone (hCG) and showed that the animals that received doses from 5 to 50 IU/day presented the highest motility values (25–35%). However, in the present study the European eels received a dose from 15 to 20 IU/day (OP-100 and OP-200 groups, respectively), and the motilities were lower than the obtained in Japanese eels. 

Furthermore, we assessed the sexual maturation at the end of the experiment using biometric parameters such as GSI and fin colour. The mean GSI value for males from OP-100 and OP-200 groups were significantly lower compared to the value reached by the control group males. However, both experimental groups showed matured and spermiating males, which means that the osmotic pump loaded with hCG_rec_ had the capacity of inducing the male’s sexual maturation. It was observed that in males that did not mature, the osmotic pump was encapsulated inside the conjunctive tissue, thus, the hormone could not be released properly. The encapsulation of the osmotic pumps inside the eels could be due to a fibrotic response by the animal’s immune system, as a measure to isolate the body from a foreign material [35]. We think that this phenomenon occurred in the present study because the male eels were very small (mean body weight of OP groups = 116.7 ± 11.1 g), compared to those in the study by Kagawa et al. [24], where they were larger (mean body weight = 336.7 ± 10.3 g) and did not have the problem of encapsulation.

Regarding the fin colour, it became darker throughout the course of maturation coinciding with previous results [30]. The results showed that the highest percentage of fish with a dark fin belonged to control group (88%), followed by OP-100 (50%) and OP-200 (33%), which seems to be related to the percentage of spermiating males in each group (control, >80%; OP-100, 50%; OP-200, 40%). Differences in the final maturation stage reached by the OP-100 and OP-200 groups may have been due to the larger size of the pump in the OP-200 group (ALZET^®^ OP-2006), which caused greater encapsulation in the males of this group.

### 4.2. Females

This is the first study obtaining sexually matured European eel females using osmotic pumps (ALZET; OP-2ML4), generating developed ovaries and even spawning events. Fifty per cent of our implanted females reached the final maturation, which is similar to the results obtained by Kagawa et al. [25] (63.4% of females). The GSI values from OP-2ML4 group (49.5 ± 2.8%) showed promising results and demonstrate that sexual maturation occurred. In Kagawa et al. [25], GSI values (27.5 ± 4.1%) were lower than in the present study. This difference may be because CPE contains higher levels of LH and FSH than SPE [36]. In addition, the amount of CPE/day released by the pumps was higher than in Kagawa et al. [25]. Therefore, the present study has proved that the osmotic pumps are an alternative method to induce the sexual maturation in European eel females, but the high variability between females poses a problem for the standardization of the method.

The female European eels need several weeks of standard hormonal treatment to reach the sexual maturation, between 16 to 25 weeks using SPE as hormonal treatment [37,38] and 12 to 25 weeks using CPE [11,39]. In the present study, sexual maturation was achieved between 13 to 17 weeks using CPE injections (the control group), which coincides with the aforementioned studies. In our study, two periods of sexual maturation were observed. A faster sexual ovulation was observed in some females in the OP-2ML4 group (50%) between 4th to 10th week, which represents the earliest sexual maturation obtained for European eel females, previously achieved by Pedersen [36], who reported ovulations between 7.5 to 11.5 weeks using a treatment of two doses of SPE per week. In addition, the weekly double dose of SPE [36] worked better than standard doses (one per week) because there was a shorter lapse of time between the injections and the levels of hormone maintained high throughout this time. In this sense, the osmotic pumps release the hormone continuously. Thus, the good results obtained in the present study coincide with the previous idea. To summarise, an in-depth study of the osmotic pumps as an alternative to hormonal injections should be necessary since the continuous hormone-releasing seems to work better than the weekly injections. Secondly, some of the implanted females reached a late sexual maturation between the 20th to 23rd week. That indicates that these females did not respond to the osmotic pumps, and they really responded to the weekly CPE injections after the 10th week. In this group it is important to note that one of the females (OP-1) showed two maturation peaks. The first one occurred in the 4th week due to the osmotic pump, while the second one occurred in the 19th week, apparently due to the CPE injections. This result shows that European eels could display a group-synchronous maturation of oocytes. Eels have been traditionally considered to have synchronous ovaries [38] because they spawn once in their life and then die. However, previous studies have demonstrated that ovaries could display a group-synchronous development when the female’s maturation is artificially induced [11,37,39,40], which agrees with the results of the present study. 

With respect to the weight evolution of the females, several studies have reported that a weight increase of 10% according to the initial weight, is a signal to induce the ovulation in female Japanese eels [41,42]. However, studies in the European eel showed that the individuals presented a higher variation, and the use of weight increment to induce the ovulation is not the more suitable method [11,36]. In the present study, the results showed a mean weight increase of 28 ± 20% (control group) and 16 ± 14% (OP-2ML4) from the beginning of the experiment. Nevertheless, this increment was lower than 10% in some of the females, and one of them even showed weight reduction. Females were fasted during the hormonal treatments, and consequently some of the animals suffered a weight reduction with respect to their initial weight. Thus, we consider that the weight gain to be examined is the one from the week previous to maturation, and not since the experiment began. In this sense, all the females showed a weight increment in comparison with the week prior to maturation. Furthermore, it would be recommended to conduct regular cannulations to assess the oocytes stage of development as a method to predict the optimal moment to induce the ovulation, which would be more accurate than the weight changes.

Concerning embryonic development, in recent years the technology and incubation methods to produce European eel larvae have advanced considerably, allowing the production of large batches of viable eggs and larvae that reach the first feeding stage [43,44,45] despite the early development stages being sensitive to biophysical parameters [46,47]. Although it was not one of the objectives of our project, some of the females that achieved maturation and developed the oocytes in the required stage were induced to final ovulation. A total of six clutches were obtained, whose embryos were incubated reaching the stage of embryo formed with head, eyes, and somites [48] as maximum. In this study, no hatching was obtained, probably due to the lack of proper incubators to maintain the culture conditions in a precise way for a correct embryonic development. 

## 5. Conclusions

With regard to European eel males, this study showed that the controlled release systems (ALZET^®^ osmotic pumps) were able to induce the maturation and spermiation, but not a sufficient amount of sperm (volume, density) with acceptable kinetic features (motility) for achieving a reasonable amount of high-quality sperm for aquaculture and research purposes.

Concerning European eel females, our study demonstrated that the use of osmotic pumps loaded with CPE was an effective method to induce sexual maturation, generating extremely early maturations (from the 4th to 10th weeks) in 50% of the females. However, the high variability between females poses a problem for using this technique for aquaculture goals, so further studies with controlled release systems must be an ongoing task for that the method standardization.

## Data Availability

Not applicable.
